# Underfeeding of Children at Blackburn

**Published:** 1905-09-23

**Authors:** 


### The Hospital.

A Journal of

The Medical Sciences and Hospital Administration.

Vol. XXXVIII.—No. 991. SATURDAY, SEPTEMBER 23, 1905.

### Underfeeding of Children at Blackburn.

The educational authorities of Blackburn have just made a very important contribution to the recent discussion on the underfeeding of school children by the publication of a report upon the subject, made after careful inquiry by Dr. Greenwood, the Medical Officer of Health for the borough. Blackburn contains 52 public elementary schools, which received, in June last, an aggregate of 22,952 scholars, among whom it was inevitable that many instances of suspected underfeeding must occur. Dr. Cameron's first proceeding was to invite the teachers to furnish him with the names and addresses of all children whom they regarded as possibly underfed, and the lists thus furnished were supplemented by various unofficial informants to whom his local knowledge enabled him to apply. In this way he obtained the addresses of 540 families, and paid a personal visit to each of them, making the necessary inquiries with tact and discrimination, and inspecting not only the children themselves, but also the general conditions of living as displayed in personal and domestic cleanliness or in its absence, and in the state of the furniture and clothing. As a first result of this procedure, he was able to eliminate 364 of the 540 houses from his inquiry, and to say positively that there was no underfeeding as far as their inmates were concerned. The occupants of the remaining 176 houses were divided into three groups: (1) those in which children might be underfed owing to permanent impoverishment of the parents, (2) those in which children might be underfed owing to temporary illness, loss of employment, or other unavoidable causes which had rendered the parents incapable of making the necessary provision, and (3) those cases in which underfeeding might be due to neglect. Inquiries were made into 13 cases under the first heading, into 114 under the second, and into 49 under the third; and the number of families in which these inquiries disclosed underfeeding was seven in the first, 69 in the second, and 19 in the third. In all cases of inquiry full details, of course omitting names and addresses, are given in the report, and the following may be taken as typical illustrations of the conditions which are described. In group 1, case 13, "Three in family, mother and two children, aged 13 and 9 years. No father. Wages 6s. weekly. There are also two old people in this house who receive 2s. 6d. weekly from the Union. Relief is often given by the Ragged School authorities and by the Union. This is a case of underfeeding." In group 2, case 19, "Five in family, father, mother and three children, aged 10, 9, and 1½ years. Father earns 18s. weekly as a labourer, but is not always in full work. The children were dirty and badly clothed. Sore heads. Rent 4s. 6d., and clubs 11d. weekly. Help has been received from the Ragged School and school teachers. There was only one small piece of bread in the house at the time of visit. I believe there is underfeeding in this family. House dirty. Furniture very poor." In group 3, case 4, "Three in family, father and two children, aged 13 and 5 years. No mother. Father out of work at present, and is addicted to drink. He is also an invalid. Rent 3s. weekly. Payments to clubs have lapsed. Relief 4s. per week. This seems to be a case of underfeeding."

The foregoing instances will serve at once as examples of the classification which Dr. Cameron has adopted. His general conclusion is so far satisfactory that he has found only 97 families in which decided underfeeding now exists, or is probable under certain conditions. These 97 families include 313 children between the ages of three and fourteen, which were made the limits of the inquiry, and it would therefore appear that the individual cases do not amount to more than 1·3 per cent. of the children in attendance at the schools. Dr. Cameron is manifestly justified in the opinion which he expresses, to the effect that, although there are certain children who require more suitable and sufficient food than they can obtain at present, the question of underfed school children in the borough is not sufficiently acute to demand direct municipal assistance. At the same time, he calls attention to the chief circumstances upon which poverty necessitating underfeeding appears to depend, and makes a variety of suggestions for promoting the effective organisation of measures calculated to diminish the evils which he describes. He divides the chief causes into those which are beyond the control of the parents (such as ill-health of the father, compelling the mother to earn money by methods which entail a lack of supervision over young children left at home, or the payment of a sum ranging from 4s. to 8s. weekly to another woman who looks after them in the absence of the parents), and those which are within their control, such as alcoholism, laziness, indifference, and bad management. He points out that considerable waste occurs in many homes through a lack of knowledge as to cheap nutritious articles of diet and economical cooking; or, in other words, that the money received might be made to "go farther" than it does, and he urges upon the education authority that the elder girl scholars should be taught the preparation and cooking of such meals as are available in a working man's home. In all this there is nothing that has not been said before, or that will not, we fear, have to be said again; but the essential novelty of the report is in its thoroughness, in its substitution of definite knowledge for vague declamation, and in the enormous help which it will render to the borough in the organisation of any systematic endeavour to remedy the evils which it displays. In these respects it is a model, and we trust that the example set at Blackburn will be extensively followed elsewhere.

