# Underfeeding of Children at Blackburn

**Published:** 1905-09-30

**Authors:** 


### EDITOR'S LETTER-BOX.

[Our Correspondents are reminded that prolixity is a great bar to publication, and that brevity of style and conciseness of statement greatly facilitate early insertion.]

### UNDERFEEDING OF CHILDREN AT BLACKBURN.

IT has been pointed out to us that in our leading article of last week on this subject an error occurred by which Dr. Cameron's name was used in place of Dr. Greenwood's. We very much regret the mistake, and apologise to Dr. Greenwood for appearing to credit his excellent work to somebody else.—ED. THE HOSPITAL.

